# Study protocol for Healthy Conversations @ Playgroup: a multi-site cluster randomized controlled trial of an intervention to promote healthy lifestyle behaviours in young children attending community playgroups

**DOI:** 10.1186/s12889-021-11789-3

**Published:** 2021-09-26

**Authors:** Stewart G. Trost, Rebecca Byrne, Kate E. Williams, Brittany J. Johnson, Anna Bird, Kate Simon, Li Kheng Chai, Caroline O. Terranova, Hayley E. Christian, Rebecca K. Golley

**Affiliations:** 1 Faculty of Health, School of Exercise and Nutrition Science, Queensland University of Technology at the Centre for Children’s Health Research (CCHR), South Brisbane, Queensland Australia; 2 Faculty of Education, School of Early Childhood and Inclusive Education, Queensland University of Technology, Kelvin Grove, Queensland Australia; 3 College of Nursing and Health Sciences, Caring Futures Institute, Flinders University, Bedford Park, South Australia Australia; 4 Telethon Kids Institute, Nedlands, Western Australia Australia; 5 Health and Wellbeing Queensland, Queensland Government, Milton, Queensland Australia

**Keywords:** Obesity, Childhood, Diet, Physical activity, Screen time, Sleep

## Abstract

**Background:**

Early childhood is a critical window for preventing obesity and chronic disease. Yet, 1 in 4 Australian children aged 5 years and under are affected by overweight or obesity; and significant proportions of children under 5 years fail to meet guidelines for diet quality, physical activity (PA), screen time, and sleep. Consequently, effective interventions to promote healthy lifestyle behaviors and prevent obesity during early childhood are needed. Community playgroups provide an opportunity for parents, carers, and children to meet in a safe and relaxed environment to play and share information. The structure, low cost and reach of playgroups provide a unique platform to engage parents in a scalable program to promote healthful lifestyle behaviors and prevent childhood obesity. However, the evidence base for the effectiveness of health promotion programs delivered in community playgroup settings is limited and lacking credible evidence from rigorously conducted randomized controlled trials.

**Methods:**

The Healthy Conversations @ Playgroup randomized controlled trial (RCT) aims to address the underlying behavioral risk factors for obesity by helping parents take effective steps to improve their child’s dietary, PA, screen time, and sleep behaviors. The intervention program comprises 10 “healthy conversations” led by a trained peer facilitator, designed to increase parents’ behavioral capability and self-efficacy to implement autonomy-supportive parenting practices. The program will be delivered biweekly during regularly scheduled playgroup sessions over 10-weeks. Effectiveness will be tested in a 2-arm cluster RCT involving 60 community playgroups in three states across Australia. After baseline assessments, participating playgroups will be randomly allocated to either intervention or wait-list control conditions. Primary outcomes (vegetable intake, discretionary foods, daily PA, screen time, sleep duration, and body mass index [BMI] z-score) will be assessed at baseline, immediately post-intervention (10-weeks; T2) and 6-months post-intervention (T3). Outcomes will be assessed for differential change at T2 and T3.

**Discussion:**

The Healthy Conversations @ Playgroup trial will rigorously evaluate a novel peer-led intervention program to promote healthful lifestyle behaviors and prevent obesity in children and families attending community playgroups. If effective, the program could be immediately scaled-up and delivered in community playgroups across Australia.

**Trial registration:**

Trial registered 22nd January 2021 with the Australian and New Zealand Clinical Trials Registry (ACTRN12621000055808).

**Supplementary Information:**

The online version contains supplementary material available at 10.1186/s12889-021-11789-3.

## Background

Childhood obesity continues to be one of the most serious health challenges worldwide. Globally, it is estimated that over 42 million children aged 0 to 5 years are affected by overweight or obesity [1]. In 2017–18, one in four Australian children aged 5 years or under were affected by overweight or obesity [2]. Of concern, young children with overweight or obesity are more likely to experience significant short-term health problems, including asthma, sleep apnoea, high blood pressure, musculoskeletal disorders, fatty liver disease, and insulin resistance [1, 3]. Compared to peers with a healthy weight, children with overweight or obesity more frequently experience bullying or teasing at school, and are at higher risks of significant mental health issues, including depression, anxiety, and disordered eating, exacerbated by weight stigma and bias [4]. In later life, they are at greater risk of adult obesity, type 2 diabetes, heart disease, certain cancers, obstructive respiratory disease, reproductive disorders, and mental health problems [1, 3]. Consequently, there is an urgent need for effective, scalable, and cost-effective public health interventions to promote healthful lifestyle behaviors and prevent chronic health conditions such as obesity during early childhood.

Healthy eating, regular physical activity, limited screen time, and adequate sleep during the early years are essential for optimal growth and development, cognitive functioning, and prevention of chronic health conditions [5]. Despite this, population-level surveys indicate that significant percentages of young children do not meet public health recommendations for diet quality, physical activity, screen time, and sleep. In Australia, almost half of 2- to 3-year-olds consume less than two servings of vegetables [6]; and discretionary foods such as sweet biscuits, cakes, and processed meats account for one-third of 2- to 3-year-old’s total daily energy intake [7]. Only 35% of 2- to 5-year-olds accumulate at least 180 min of daily physical activity, [8] while 74% of 2- to 4-year-olds exceed the screen time recommendation of less than 1 h per day [9]. Up to 40% of Australian parents report that their infants and young children experience sleep problems and between 10 and 20% of preschool children do not meet current sleep guidelines of 11 to 14 h per 24-h period [10]. Similar trends have been reported in other countries [11].

Intervention studies conducted in home, childcare, and health service settings have demonstrated that obesity-related behaviors can be improved via the promotion of autonomy-supporting parenting practices, and that the rate of change in body mass index (BMI) can be slowed [12]. However, the evidence base is incomplete and substantial gaps in knowledge remain. First, intervention studies conducted to date have primarily targeted obesity-risk behaviors in infants or pre-schoolers, but not toddlers. In a systematic review of obesity prevention trials in children 0 to 5 years, only six of the 23 intervention studies included toddlers (18–36 months) of which one was judged to be of high quality, and none reported significant reductions in BMI [13]. Second, interventions to prevent obesity during early childhood have largely ignored the contribution of sleep to maintaining a healthy weight, despite consistent evidence linking short sleep duration to excess adiposity in children [14, 15]. Third, very few obesity prevention programs have been delivered in existing community-based parent groups where parents have already formed social networks and can learn from and support each other [12].

One such setting, are community playgroups. Community playgroups provide an opportunity for parents, carers, and children to meet in a safe and relaxed environment to play and share information [16]. Open to all families with children under school age, community playgroups present a low-cost, light-touch family support platform because they are run by volunteer parents within a local area, at minimal charge to attending families. Typically, playgroups meet for 2 h per week in a local community venue, sharing unstructured indoor and outdoor play and art/craft activities. Within the early childhood service system in Australia, playgroups form an important, largely universal, bridge between the maternal child health care system and formal early childhood education and care services. They are distinct from other early childhood services because a parent or carer attends with the child and remains responsible for the child during the session. Thus, community playgroups represent an ideal setting to engage parents and deliver programs and services to enhance family functioning and promote child health and wellbeing. They also offer considerable feasibility and efficiency in terms of delivering evidence-based early childhood obesity prevention programs. Community playgroups exist in over 80% of all Australian postcodes in metropolitan, regional, rural, and remote areas [16, 17]. Recent national data indicates that over 50% of children aged 2–3 years attend a playgroup [18].

The structure, low cost and reach of playgroups provide a unique platform to engage parents in a scalable program to promote healthful lifestyle behaviors and prevent childhood obesity. However, the existing evidence base for the effectiveness of health promotion programs delivered in community playgroup settings is limited and lacking credible evidence from rigorously conducted randomized controlled trials. In a systematic review of health promotion programs delivered in playgroups [19], only five publications included nutrition, physical activity, or screen time outcomes, and none focused on sleep. All studies were of low methodological quality, relying on single group pre-post designs and/or qualitative analysis of interviews with playgroup facilitators. Consequently, there is very limited scientific evidence to inform policy makers, service planners, and playgroup facilitators about what works and what does not work when it comes to promoting healthy lifestyle behaviors in playgroup settings.

To bridge this knowledge gap, the Healthy Conversations @ Playgroup study will implement and rigorously evaluate a novel peer-led intervention to promote healthful lifestyle behaviors and prevent obesity in children and families attending community playgroups. The primary aims are: 1) evaluate the effectiveness of a 10-week healthy conversations intervention to improve dietary, physical activity, screen time, and sleep behaviours in children aged 0–5 years; and, 2) examine the impact of the intervention on child BMI z-score at 6-months post-intervention. As secondary aims, the effects of the intervention on parenting practices related to child obesity related behaviors will be examined. It is hypothesised that children in intervention playgroups will exhibit significantly better post intervention diet quality, physical activity, screen time, and sleep behaviors than children attending wait-listed control playgroups. We further hypothesize that children in playgroups allocated to the intervention will exhibit significantly lower BMI z-scores at 6-months post intervention than children attending wait-listed control playgroups.

## Methods

### Study design

To address the study aims, we will conduct a multi-site cluster randomized controlled trial involving 60 community playgroups operating in three states across Australia – Queensland (*n* = 20), South Australia (*n* = 20), and Western Australia (*n* = 20). After baseline assessments, participating playgroups will be randomly allocated to either the intervention or wait-list control conditions. Primary study outcomes will be assessed for differential change immediately post intervention (10-weeks; T2) and 6-months post intervention (T3). An overview of the study design, the schedule for enrolment and study assessments is shown in Table 1.

**Table 1:**
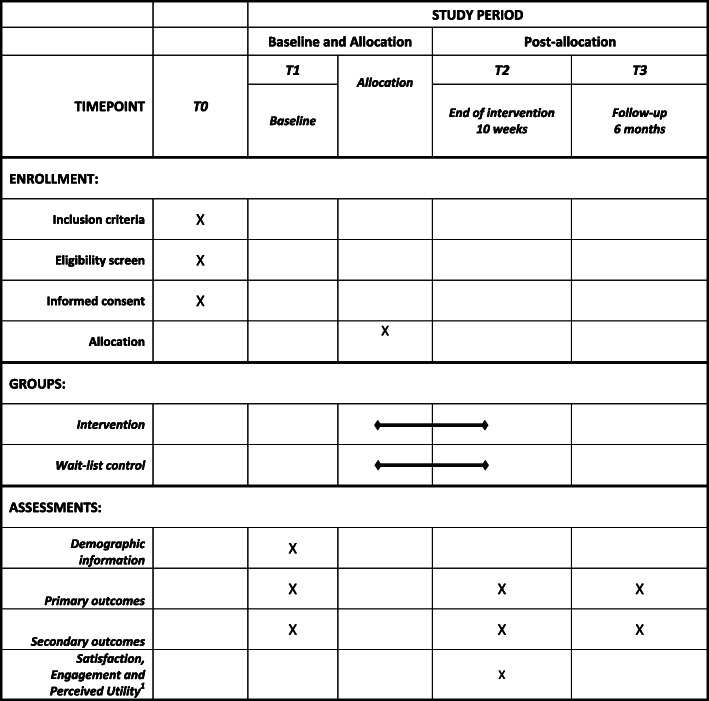
Schedule of enrolment, intervention, and assessments

Primary outcomes: daily servings of vegetables, daily servings of discretionary foods, percentage of time in moderate-to-vigorous p20hysical activity (MVPA), daily screen time, and sleep duration per 24 h period

Secondary outcomes: parental self-efficacy, parenting practices related to healthy eating, physical activity, screen time, and sleep

^1^ Provided only to parents allocated to the intervention group

### Eligibility criteria

Playgroups meeting the following inclusion criteria will be eligible to participate in the program: located within a 150 km radius of the Queensland Centre for Children’s Health Research, Flinders University, South Australia or Telethon Kids Institute, University of Western Australia; operating as a community playgroup (that is, coordinated by parent volunteers rather than a paid facilitator); and at least four parent-child dyads willing to participate in the evaluation of the program. In each participating playgroup, all parents with children between the ages of 1 and 5 years will be invited to participate in the evaluation. Parents with multiple children within the specified age range will be asked to enroll their oldest child in the study. Parents will be excluded if they are unable to give informed consent or have insufficient literacy to complete data collection. Parents with children outside the target age range or children with chronic health conditions that may adversely influence weight status, physical activity, diet, or sleep (e.g., severe respiratory disease, congenital heart disease), will be able to participate in the program, but will be excluded from the evaluation.

### Recruitment

All eligible community playgroups will receive information about the Healthy Conversations @ Playgroup program through their respective state-level playgroup association via social media, newsletters, email or flyers. Playgroups registering an interest in the program will be contacted by a member of the research team to further explain the project and obtain permission to visit the playgroup. If permission is granted, a research team member will attend a regular playgroup session and explain the project to all parents in attendance. Playgroups will then be given time to discuss and decide if they, as a group, would like to receive the program. If the playgroup elects to receive the program, project staff will initiate the necessary informed consent procedures. Prior to participation, full written and informed consent will be obtained from legal parents/guardians. Parents/guardians will have the option to sign a hard copy of the consent form at the session or use a QR code to access the consent form on-line and sign it electronically. This process will continue until the required number of playgroups with sufficient participants in each site are recruited.

### Sample size and power

For Aim 1, the intervention effect is the net difference for change in the healthy lifestyle behaviors immediately post intervention and at 6-months follow-up. The research hypothesis is that the intervention will result in net differences of 0.5 serving of vegetables (38 g per day), 1.5 servings of discretionary foods per day, 15 min or 2% of time in MVPA, 30 min screen time per day, and 40 min of sleep per 24 h period. The null hypothesis is a net difference of zero. For Aim 2, the intervention effect is the net difference for change in BMI z-score at 6-months follow-up. The research hypothesis is that the intervention will result in a difference of 0.30 BMI z-score units. The null hypothesis is a net difference of zero. For a 2-year-old at the 84th percentile or a BMI z-score of 1.0, a difference of 0.30 z-score units equates to a clinically meaningful reduction of 0.4 kg in excess weight.

Table 2 reports minimal detectable differences for samples of 20, 30, and 40 community playgroups per condition. All calculations were performed assuming a power of 0.80, a 2-tailed alpha level of 0.05, an average cluster size of six parent-child dyads, and an intra-cluster correlation (ICC) of 0.10 and 0.02 for the child health behaviors and BMI z-score, respectively. Standard deviation estimates for vegetable intake were based on dietary intake data from the NOURISH randomized controlled trial [20]. The standard deviations for time in physical activity and screen time were based on previously published studies in Australian children aged 0–5 years [21]. The standard deviation for sleep duration was based on published data on Australian children [10]. The standard deviation for change in BMI z-score was based on data from the NOURISH randomized controlled trial [20]. Based on these calculations, a sample of 30 playgroups per condition achieves ≥80% power to detect the hypothesized between-group differences for each of the primary outcomes. To offset a projected attrition rate of up to 25%, an additional two children within each playgroup will be recruited, providing an overall sample of 60 community playgroups and 480 parent-child dyads.

**Table 2:** Minimal detectable differences for child health behaviors and BMI z-score

Study Outcome	Hypothesised Effect	Est. SD	20 Playgroups (***n*** = 120)	30 Playgroups (***n*** = 180)	40 Playgroups (***n*** = 240)
Veg Intake	½ serve, 38 g/d	58 g/d	26 g/d	21 g/d	18 g/d
Discretionary Foods	1.5 serv/d	2 serv/d	1 serv/d	0.7 serv/d	0.6 serv/d
% Time in PA	2.0%	5%	2.2%	1.8%	1.6%
Screen Time	30 min/d	75 min/d	33 min/d	27 min/d	24 min/d
Sleep Duration	40 min/d	96 min/d	43 min/d	35 min/d	30 min/d
BMI z-score	0.30	0.90	0.34	0.28	0.24

### Randomization, allocation, and blinding

Group assignment for each site will be completed by an independent statistician using an allocation sequence generated by permuted block randomization. After baseline assessments, playgroups in each site will be randomized to the next condition (intervention or wait-list control) on the list using the database randomization module in the Research Electronic Data Capture (REDCap) platform hosted at The Queensland University of Technology [22]. Trained assessors blinded to group allocation will collect the primary and secondary outcome data. Within a health behavior intervention, it is not possible to blind either the persons delivering the intervention (peer facilitators – see below) or participants; however, peer facilitators will not take part in outcome assessments.

### Healthy conversations @ playgroup intervention

The Healthy Conversations @ Playgroup program aims to address the underlying behavioral risk factors for obesity and chronic disease by helping parents take effective steps to improve their children’s dietary, physical activity, screen time, and sleep behaviors. Grounded in Social Cognitive Theory [23] and Self-Determination Theory [24], the intervention comprises 10 “healthy conversations”, led by a peer facilitator, designed to increase parents’ behavioral capability and self-efficacy to implement autonomy-supportive parenting practices related to healthy eating, physical activity, screen time, and sleep. The intervention utilizes Behavior Change Techniques to change parenting health behavior practices, as classified the Behavior Change Technique Taxonomy v1 [25]. Behavior change techniques include goal setting (behavior), problem solving, action planning, review behavior goal(s), social support (unspecified), instruction on how to perform a behavior, behavior substitution, and verbal persuasion about capability (Additional file 1). The program will be delivered biweekly, on-site during regularly scheduled playgroup sessions by trained peer facilitators over a 10-week period (which coincides with the Australian public-school term). Informed by the results of focus groups conducted in community playgroups [26], the intervention addresses five major themes, which are described in Table 3.

**Table 3:** Description of the five healthy conversation topics and key messages

Healthy conversation topics	Key messages
***Session 1 – Reducing stress at mealtimes***	
Food refusal and child hunger/fullness	“Parent provides, child decides”: Parents can provide healthy meals and snacks; and let the child decide if they are hungry.
Supporting child taste development	Offering new or disliked foods multiple (10–12) times helps children taste and accept (or even like) new foods.
***Session 2 – Limiting screens without tantrums***	
Create a family digital media plan	Creating a ‘family technology plan’ helps everyone in your family balance and manage technology use.
Alternatives to screens	Limiting screen time is challenging so it helps to plan ahead and make a “toolbox” of alternatives.
***Session 3 – Supporting movement skills in children***	
Get children moving with active play	For young children, physical activity is about active play. It does not have to involve structured or planned activities.
When it comes to encouraging active play, parents make a difference	Parents make a difference – encourage your child to be active by watching them, participating with them, and helping them discover activities they like to do.
***Session 4 – Bedtime activities and routines to support sleep***	
Creating a bedtime routine	A consistent bedtime routine can help children sleep.
Overcoming barriers to bedtime routines	Make the bedroom environment as “sleep friendly” as possible and enlist the support of “significant others” in implementing sleep routines.
***Session 5 – Celebrating achievements***	
Wins with food refusal and child taste development	Be confident in yourself and your ability as a parent to positively influence your child’s health behaviors.
Wins with screen time, active play, and sleep	Keep up the good work and be kind to yourself!

### What is a healthy conversation?

A healthy conversation takes place opportunistically between parents attending the regularly scheduled playgroup. With the guidance of a trained peer facilitator, parents are encouraged to share and reflect on parenting challenges related to healthy eating, screen time, physical activity, and sleep with a view towards making small but important changes to improve their child’s health. The conversation typically concludes with signposting to follow-up support services or information. Initially developed for use in health care settings [27], the healthy conversations approach has been successfully implemented in other settings. Most notably, this approach has been used by childcare workers in the United Kingdom to improve diet and physical activity in families experiencing socioeconomic disadvantage [28].

### Peer facilitators

The intervention will be delivered by trained peer facilitators. Peer facilitators will not be researchers or health professionals, but parents who have training and/or experience in communications and group facilitation. In our pilot study, this approach was shown to be more feasible than training a volunteer parent from each community playgroup [29]. Facilitator training will involve the equivalent of a full day face-to-face skills-based workshop (hybrid in-person and video link) and biweekly video link peer support meetings to check-in and assist with fidelity maintenance across the three sites. Through skill-based learning strategies, problem-solving and role-plays, peer facilitators will be taught how to: 1) deliver each component of the intervention; 2) effective facilitation skills; 3) identify and overcome common barriers to program implementation; and 4) use of social media for signposts. Prior to the workshop, peer facilitators will receive a Facilitator Handbook (hard copy and electronic) which provides a complete list of open-ended questions for conversation starters, suggestions for open-ended discovery questions and prompts, suggested responses to parent comments, suggestions for wrapping up the conversation and goal setting, and copies of the social media signpost.

### Implementing healthy conversations at playgroup

During each program session, the peer facilitator will lead two 10- to 15-min healthy conversations related to a specific theme. The facilitator opens the discussion with a question for the group (conversation starter) and then guides the discussion using open-ended questions and prompts. Parents are encouraged to reflect on what they currently do as parents – the actions, behaviors and strategies they use to encourage, discourage, support, or manage their child’s health behaviors. Parents are encouraged to think about “what works” for them and “what they could do differently” to improve health behaviors. Through group discussion, parents collectively formulate ideas and strategies to deal with specific challenges. When necessary, the facilitator provides ideas to keep the conversation moving and on track. At the end of the discussion, the facilitator encourages parents to select a clear and achievable strategy from those discussed in the conversation to implement at home.

### Signposting intervention

Between each session, parents will receive ‘signposts’ directing them to information and support about the topics covered. These signposts will be delivered via social media, using private Facebook groups set up for the purpose of the program (one per site to align with differing school term dates between sites). Project staff will post content to the Facebook group two times between each session. Each post will be related to the most recent conversation topics, and may include videos, links to articles and prompts to action. The aim is to provide participants with further information on the topic, prompt action in relation to the health behavior discussed at the previous session, encourage sharing of experiences, and increase social support. Facilitators will moderate posts (e.g., remove posts, pin, or unpin posts) and respond to comments posted by parents.

### Wait-list control

The intervention playgroups will be compared to a wait-list control group completing their usual playgroup sessions. Playgroups allocated to the wait-list control group will be offered the 10-week program at the conclusion of the 6-month follow-up assessment.

### Participant characteristics

Demographic information will be collected at baseline using an online survey and will include child date of birth, child sex, parental age, parental education, and family structure.

### Primary outcomes

#### Dietary intake

Children’s dietary intake will be reported by parents using a short questionnaire developed to assess obesity-related dietary behaviors in 1- to 5-year-olds [30]. The 13-item questionnaire asks parents to report how many times over the past 7 days their child has consumed vegetables (2 items), sweetened drinks (e.g., fruit juice, cordial/squash, soft drinks/soda, flavoured milk; 2 items) and discretionary foods (e.g., chocolate, potato crisps or savory biscuits, processed meat, sweet baked goods, takeaway foods; 7 items), as well as type of milk (1 item) and bread (1 item) consumed. The short dietary questionnaire has been shown to have acceptable relative validity (Spearman’s rho = 0.62 to 0.89) and test-retest reliability (ICC = 0.80–0.97) [30].

#### Physical activity

The percentage of daily time spent in moderate-to-vigorous intensity physical activity (MVPA) will be measured using the Axivity AX3 accelerometer (Axivity Ltd. Newcastle, United Kingdom). Participants will receive the accelerometer and an information pack via express mail. The pack will include an introductory letter to parents, a detailed instruction sheet, an activity monitoring log sheet to record any activity monitor removals, and a pre-paid padded envelope to return the accelerometer and log sheet to the research team. The child will be asked to wear the accelerometer on their non-dominant wrist 24-h per day for seven consecutive days (except for bathing or water-based activities).

Upon return of the accelerometer via express mail, raw accelerometer data (50 Hz) will be downloaded and processed into physical activity metrics using a random forest physical activity classification algorithm specifically developed for children under five [31]. This validated machine learning algorithm uses 20 features extracted from the raw tri-axial acceleration signal to classify activity type and quantify daily time spent in sedentary activities (sitting or lying down), light-intensity activities and games (slow walking, standing, standing arts and crafts), walking, running, and moderate-to-vigorous intensity activities and games (active games with balls, riding bikes/scooters). In a free-living evaluation, the random forest algorithm exhibited an overall classification accuracy of greater than 80%. MVPA will be calculated by summing daily time spent in walking, running, and moderate-to-vigorous activities and games.

#### Screen time

Children’s screen time will be measured using four items from the Movement Behaviour Questionnaire (MBQ). Parents will report the amount of time their child spends: watching television programs, videos/internet clips or movies on a television, computer, or portable/mobile device such as tablet or smartphone; and, playing games, looking at photos, or video chatting on a screen-based device such as a computer or laptop, videogame console, iPad, tablet, or smartphone. Responses will be recorded for a typical weekday and weekend day over the past week. To estimate sedentary screen time, parents will report the amount of time their child was using screens while standing or being active. Sedentary screen time will be calculated by subtracting “active” screen time from total screen time. Weekday and weekend responses will be averaged to estimate total screen time and sedentary screen time. The screen time estimates from the MBQ have been shown to exhibit excellent test-retest reliability (ICC = 0.91 to 0.93) [30].

#### Sleep

Sleep duration over the previous week will be parent reported using three items from the MBQ. These items assess typical day and night sleep duration and regularity of evening sleep routine. Sleep duration and regularity responses from the MBQ have been shown to exhibit excellent test-retest reliability (ICC 0.85–0.95) [30]. Sleep duration and quality will also be measured using data from the AX3 accelerometer. Raw acceleration signal from the wrist will be scored for sleep-wake state using the algorithm developed by Van Hees and colleagues [32].

#### Body mass index and weight status

Height and weight will be measured using a standard protocol. Height will be measured to the nearest 1 mm using a calibrated portable stadiometer (Seca 213, Hamburg, Germany)). Weight will be measured to the nearest 0.1 kg using a calibrated portable digital scale (Tanita Corporation, IL, USA). BMI will be calculated as body weight in kilograms divided by height in meters squared. BMI will be converted to age- and sex-specific percentiles or z-scores using the World Health Organization Growth Standards for 0 to 5 years [33].

### Secondary outcomes

#### Parental self-efficacy

Parental confidence in relation to promoting healthy eating, reducing sedentary time, and promoting physical activity will be assessed using an established tool from the Infant study [34]. The scale comprises 20 items addressing four parenting domains: 1) self-efficacy for promoting healthy eating; 2) self-efficacy for limiting non-core foods; 3) self-efficacy for promoting physical activity; and 4) self-efficacy for limiting screen use. Responses are recorded on a 5-point scale with endpoints ranging from ‘not at all confident’ to ‘extremely confident’. These scales have established evidence of validity and reliability [34], with internal consistency ranging from 0.72 to 0.85 [35].

#### Parent feeding practices

The Feeding Practices and Structure Questionnaire (FPSQ-28) [36] will be used to measure the following two feeding constructs: ‘Reward for Eating’ and ‘Persuasive Feeding’. Items are scored on a 5-point Likert scale with higher scores indicating greater endorsement of that practice. The FPSQ-28 has been validated with mothers of young children (2–5 years) assessing the effects of an intervention on feeding practices. The two constructs selected for this study show good internal consistency: ‘Reward for Eating’ α = 0.89 and ‘Persuasive Feeding’ α = 0.73 [36].

#### Parental support for physical activity

Parental support for children’s physical activity will be assessed using a 5-item scale developed by Trost et al. [37]. Parents report how often they ‘encourage their child to do physical activities or play sports’; ‘play outside or do physical activity or sports with their child’; ‘provide transportation to a place their child can do physical activity or play sports’; ‘watch their child participate in sport, physical activities or outdoor games’; and, ‘tell their child that physical activity is good for his or her health’. Responses are recorded on a 6-point scale with endpoints ranging from zero (never) to five (daily). In a recent study of Australian 3- to 6-year-olds, the internal consistency of the scale, as measured by Cronbach’s alpha, was acceptable at 0.79 [38].

#### Screen time parenting practices

Two scales from the Physical Activity and Screen Time Parenting Practices Questionnaire [39] will be used to measure screen-related parenting practices: “limiting or monitoring screen time” and “use of screen time to reward/control child behavior.” For the limiting or monitoring screen time scale, parents will rate statements describing how tightly they monitor their child’s screen time on weekdays and weekend days. Responses are recorded on a 5-point scale with endpoints ranging from ‘strongly disagree’ to ‘strongly agree’. For the use of screen time to reward/control child behavior scale, parents report how often they use screen time as a reward for good behavior; take away screen time as punishment for bad behavior; offer additional screen time as a reward; and use screen time to control children’s behavior. Responses are recorded on a 6-point scale with endpoints ranging from ‘never’ to ‘very often’. Both scales have evidence of internal consistency (α = 0.85 to 0.89) and test-retest reliability (ICC = 0.95 to 0.97) [40].

#### Sleep parenting practices

Parenting practices related to sleep will be measured using the consistency scale (5 items) from the Bedtime Routines Questionnaire [41]. Parents will be asked to rate how often: their child slept in the same place, went to bed at the same time; was put to bed by the same person; performed the same activities before going to bed; and performed events in the same order before going to bed. Responses are recorded on a 5-point scale with endpoints ranging from ‘almost never’ to ‘nearly always. The bedtime consistency scale has established evidence of internal consistency (α = 0.79 to 0.82) and test-retest reliability (ICC = 0.86) [40].

### Intervention Fidelity

Following each playgroup session in which the intervention is delivered, the facilitator will complete a checklist documenting the essential elements of the program. The checklist will record overall parent attendance, the number of parents engaged in each conversation, approximate time spent in each conversation, the extent to which key messages were delivered as planned, and parents’ level of engagement in the conversations. Facilitators will be able to note the highlights of the session as well as any challenges encountered during the session.

### Overall satisfaction, engagement and perceived utility

At the conclusion of the intervention program, parents will be invited to complete a survey assessing overall satisfaction with the Healthy Conversations @ Playgroup program, engagement with the program, enjoyment of the conversations, and the perceived utility of the group conversations and Facebook posts. Parents will be asked to rate on a 5-point scale their overall satisfaction with the program (not at all satisfied, slightly satisfied, moderately satisfied, very satisfied, extremely satisfied); the usefulness of each of the five healthy conversation topics and associated Facebook posts (not at all, a little, somewhat, mostly, very); how frequently they took part in the conversations (always, usually, about half the time, seldom, never); and enjoyment of the conversations (not at all, a little, somewhat, mostly, a great deal). Parents will also be asked if they would recommend the Healthy Conversations @ Playgroup program to others and comment on their response.

### Statistical analysis

Statistical analyses will follow standard principles for RCTs using two group comparisons including all participants on an intention-to-treat basis. Between-group differences in the primary and secondary outcomes will be tested using a general linear mixed model, which accounts for the clustering of parent-child dyads within playgroups. Within each model, condition, time, the condition by time interaction will be included as fixed effects, with playgroup nested within condition, parent-child dyads nested within playgroup and condition, and the playgroup by time interaction nested within condition included as random effects.

### Ethics

Ethical approval has been granted from the Human Research Ethics Committee of Children’s Health Queensland (HREC/19/QCHQ/66486) and administratively reviewed by The Queensland University of Technology (2000000576), Flinders University (2586) and The University of Western Australia (RA/4/20/6386). The trial has been prospectively registered with the Australian New Zealand Clinical Trials Registry (ACTRN12621000055808). Before enrolling in the trial, full written and informed consent will be obtained from legal parents/guardians. Participant data will be managed on a secured electronic database (REDCap) and hardcopy forms stored securely at the research facility.

## Discussion

As concerns for the health of young children and the resultant long-term consequences increases, light-touch universal interventions with the potential for efficient scalability and high reach are needed. In Australia, playgroups have strong engagement and acceptance within the community, but to date have been an underutilised resource in terms of health promotion. The Healthy Conversations @ Playgroup program involves providing parents the knowledge, skills, and confidence to create and manage environments for their children in which healthy behaviors across multiple domains can develop and flourish. The intervention is delivered in an ecologically-valid setting in which parents are already engaged, and likely to be open to learning through a peer facilitator. Importantly, in supporting parents as the main mechanism for change, the intervention has the potential to impact multiple children in each family setting. Through social-cognitive learning approaches, its impact may be even more broad as involved parents take on new knowledge and strategies and share them with others in the playgroup even after the research has ceased. Developmentally, supporting parenting-for-healthy-behaviors in the years prior to school is critical as it is in this time period that parents have the highest opportunity to influence their children’s lifestyle habits in ways that will support positive longer-term health trajectories.

## Supplementary Information

**Additional file 1: Table 1.** Behavior change techniques used in the Healthy Conversations @ Playgroup intervention program.

## Data Availability

Not applicable.

## Abbreviations

BMI: Body mass index
FPSQ: Feeding Practices and Structure Questionnaire
Hz: Hertz
ICC: Intra-cluster correlation
MVPA: Moderate-to-vigorous physical activity
MBQ: Movement Behaviour Questionnaire
PA: Physical activity
RCT: Randomized controlled trial
REDCap: Research Electronic Data Capture
