# Supplementary material for: Study protocol for Healthy Conversations @ Playgroup: a multi-site cluster randomized controlled trial of an intervention to promote healthy lifestyle behaviours in young children attending community playgroups
Source: BMC Public Health. 2021 Sep 26;21:1757. doi: 10.1186/s12889-021-11789-3 (PMC8474833; doi:10.1186/s12889-021-11789-3)
Supplement: Supplementary file 1 — Additional file 1: Table 1. Behavior change techniques used in the Healthy Conversations @ Playgroup intervention program. [file 12889_2021_11789_MOESM1_ESM.docx]

Additional file 1.

Additional Table 1: Behavior change techniques used in the Healthy Conversations @ Playgroup intervention program.

| Components of the intervention | Behavior Change Technique^1^ | Application of Behavior Change Techniques |
| --- | --- | --- |
| Peer facilitated fortnightly healthy conversations at Playgroup  Provision of weekly private Facebook group signposts related to conversation topics | 1.1 Goal setting (behaviour)  1.2 Problem solving  1.4 Action planning | Parents are encouraged to discuss barriers and possible solutions  Parents are encouraged to select one strategy they will try at home that captures the goal and action plan |
|  | 1.5 Review behavior goal(s) | Facebook signposts prompt parents to review process on their goals for the fortnight  The final conversation session encourages parents to reflect on their goals over the program |
|  | 3.1 Social support (unspecified) | Throughout the conversation sessions and Facebook signposts parents are encouraged to support each other in changing behavior, by sharing ideas and progress |
|  | 4.1 Instruction on how to perform a behavior | Conversation sessions include brief guidance on how to support child health behaviors (e.g. bedtime routine)  Facebook signposts included links to parenting resources that provide further strategies (e.g. how to develop a family technology plan) |
|  | 8.2 Behavior substitution | Parents are encouraged to swap less favorable behaviors and habits for those that support child’s healthy behavior development |
|  | 15.1 Verbal persuasion about capability | The final conversation session focuses on persuading parents they have the ability to positively influence their child’s health behaviors |

^1^ Behavior Change Techniques coded from the BCTTv1 from Michie et al. 2013 [25].
